# Effect and mechanism of transient receptor potential canonical channel 3 on hyperoxic lung injury in neonatal rats

**DOI:** 10.1002/pdi3.65

**Published:** 2024-03-07

**Authors:** Xingmeng Fu, Xiaoxia Gong, Tianyi Wu, Lirou Chen, Zhou Fu, Chang Shu

**Affiliations:** ^1^ Department of Respiratory Children's Hospital of Chongqing Medical University Chongqing China; ^2^ National Clinical Research Center for Child Health and Disorders Chongqing China; ^3^ Ministry of Education Key Laboratory of Child Developmental and Disorders Chongqing China; ^4^ Key Laboratory of Pediatrics of Chongqing Chongqing China

**Keywords:** bronchopulmonary dysplasia, calcium ion channels, TRPC3, TRPC3 agonists

## Abstract

The aim of this study is to research the expression of the transient receptor potential canonical channel 3 (TRPC3) in a neonatal hyperoxic lung injury model of bronchopulmonary dysplasia (BPD), and to further investigate the role of the TRPC3/nuclear factor‐κB (NF‐κB) signaling pathway in hyperoxia‐induced BPD by a TRPC3 agonist (GSK1702934A). The hyperoxic lung injury model of BPD was established in Sprague–Dawley neonatal rats. Hematoxylin and eosin (HE) staining and radial alveolar count (RAC) values showed that the hyperoxic lung injury model of BPD was successfully established in the neonatal rats, and pulmonary edema was found in the neonatal rats with BPD. The results of reference transcriptome sequencing, Quantitative real‐time PCR (qPCR), and western blot showed lower pulmonary expression of TRPC3 in the BPD group than in the control group. Immunofluorescence showed predominant expression of TRPC3 in airways and pulmonary vessels, and the fluorescence intensity of the BPD group was lower than that of the control group. Lung dry‐to‐wet weight ratio, HE staining, and RAC value showed that the lung histomorphology significantly improved in the BPD + TRPC3 agonist group compared with the BPD group on day 14 but did not revert to the level of the control group. According to qPCR results, compared with the control group, the expression of NF‐κB1 decreased and the expression of NF‐κBiz increased in the BPD group, whereas the expression of NF‐κBiz decreased in the BPD + TRPC3 agonist group. Therefore, we draw the conclusion that TRPC3 may activate NF‐κB by inhibiting NF‐κBiz to promote cell proliferation and lung growth and development.

## Graphical abstract

TRPC: ion channels mediates oxidative stress response, participates in airway mechanical traction and acute lung inflammation, and is widely expressed in lung tissue. Transient receptor potential channel 3 may regulate the NF‐κB signaling pathway to affect cell permeability and cell proliferation by mediating calcium influx.

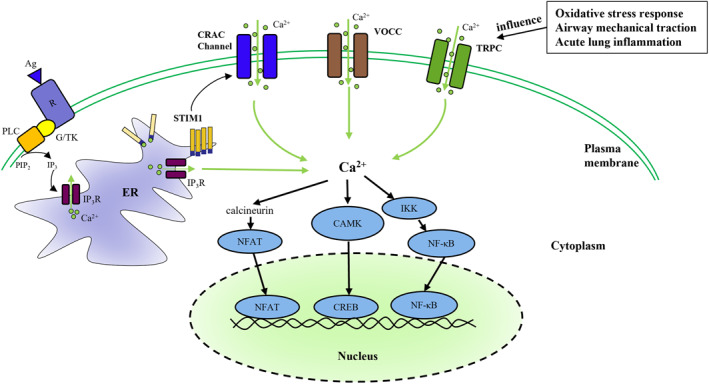

## 1 INTRODUCTION

Bronchopulmonary dysplasia (BPD) is one of the most serious sequelae of premature birth and the most common form of infantile chronic lung disease.^1^ In recent years, with the development of perinatal medicine and the establishment of neonatal intensive care units in various regions, the survival rate of very‐low‐ and ultra‐low‐birth‐weight infants has increased significantly, resulting in a rising incidence of BPD, which increases with the decrease in gestational age.^2^, ^3^

Bronchopulmonary dysplasia is the main cause of chronic respiratory disease in infancy, and it seriously affects the infants' survival rate and quality of life. According to relevant data,^4^ the fatality rate of severe BPD is 25%, of which 10% occurs in the first year. The main causes of death are repeated lower respiratory tract infections, persistent pulmonary hypertension, pulmonary heart disease, and sudden death.^4^ Our previous study^5^ showed that 121 children with BPD were readmitted 242 times for lower respiratory tract infections within the first 2 years and that the BPD patients showed significant airflow limitation and lung function impairment in adolescence and adulthood.

Bronchopulmonary dysplasia is the result of an abnormal repair response to prenatal and postnatal damage to the developing lung.^6^ Although the pathogenesis of BPD is still insufficiently clear, it likely involves a variety of prenatal and postnatal mechanisms, including preterm birth, genetic susceptibility, oxidative stress, stress injury, and infection.^6^, ^7^

The regulation of intracellular Ca^2+^ homeostasis is a key factor in many physiological processes.^8^ Changes in Ca^2+^ homeostasis in vascular endothelium and smooth muscle are involved in the pathophysiological processes in the pulmonary vascular system, and Ca^2+^ signaling in endothelial cells is essential for maintaining the integrity of the endothelial barrier.^9^ Changes in Ca^2+^ concentration play a key role in regulating the contraction, migration, and proliferation of vascular smooth muscle cells.^10^

Transient receptor potential (TRP) channels is an important nonselective cation channel superfamily located on the cell membrane. The main ions that pass through TRP channels including Na^+^, Ca^2+^, and Mg^2+^.^11^ TRP channels are divided into six families, namely TRPC (canonical), TRPV (vanilloid), TRPM (melastatin), TRPA (ankyrin), TRPP (polycystin) and TRPML (mucolipin).^12^

The ion channels of the pulmonary vascular system—transient receptor potential canonical channel (TRPC) subfamily members—mainly allow the entry of Na^+^ and Ca^2+^, and contribute to mediating the calcium store‐operated Ca^2+^ entry (SOCE) pathway and the receptor‐activated Ca^2+^ entry (ROCE) pathway.^13^ Except for TRPC2, which is not detectable in humans, other TRPC channels are widely expressed in human and animal pulmonary artery smooth muscle cells (PASMC), pulmonary artery endothelial cells, airway smooth muscle cells (ASMC), and bronchial epithelial cells.^14^ Transient receptor potential canonical channel 3 (TRPC3) is the main representative of nonselective cation channels in human ASMC, while TRPC3 and TRPC6 are related to the proliferation of PASMC.^14^, ^15^

TRPC1 and TRPC4, and TRPC3 and TRPC6 can form isomers, which regulate the barrier function in pulmonary microvascular endothelial cells.^16^ Activation of TRPC3/6 promotes the loss of endothelial barrier function in acute pneumonia, but TRPC3/6‐mediated signaling pathways may also be important for maintaining endothelial barrier integrity.^16^ It has been shown that TRPC may be involved in airway mechanical stretch as a mechanosensitive stretch activation channel, which can be activated not only by phospholipase C (PLC)‐coupled receptors, but also by plasma‐membrane thinning induced by mechanical stimulation.^17^, ^18^ It has also been shown that TRPC3 can form redox‐sensitive cation channels.^19^ The TRPC3 channel is also associated with diseases such as asthma,^20^ pulmonary hypertension,^21^ and chronic obstructive pulmonary disease.^22^ It has been shown that the expression level of TRPC3 increases with the proliferation of ASMCs, and inhibition of TRPC3 can significantly reduce the proliferation and migration of ASMCs.^20^ However, it is not yet clear whether the TRPC3 channel is involved in the occurrence and development of BPD.

Hyperoxic injury, mechanical ventilation, premature delivery, infection, and other factors may lead to the occurrence of BPD, resulting in the simplification of alveoli and pulmonary vessels. TRPC3 mediates oxidative stress response, participates in airway mechanical traction and acute lung inflammation. TRPC3 is widely expressed in lung tissue, where it affects cell permeability and cell proliferation. Therefore, we hypothesized that in a neonatal hyperoxic lung injury rat model of BPD, the simplification of alveoli and pulmonary blood vessels would reduce the expression of TRPC3 in the lungs, and the decreased TRPC3 expression would impair cell membrane permeability and inhibit cell proliferation, thereby promoting the development of BPD and participating in the regulation of BPD through the nuclear factor‐κB (NF‐κB) signaling pathway.

## 2 MATERIALS AND METHODS

### 2.1 Animal model

Sprague–Dawley (SD) rats were purchased from the Animal Experimental Center of Chongqing Medical University, following the guidelines for the use of experimental animal care issued by the National Institutes of Health and approved by the experimental ethics committee of Children's Hospital of Chongqing Medical University. The neonatal SD rats within 24 h after birth were randomly divided into the control group and the BPD group. The rats from the BPD group were placed in a closed cage with continuous oxygen input for 14 days, and oxygen concentration was monitored digitally to ensure its maintenance at 75%–85%. The newborn rats in the BPD group were placed with the dam in the BPD group to ensure that the newborn rats were breastfed and their body temperature was maintained. And every 48 h, we exchanged the dams of the normal group and the BPD group to prevent maternal death. The ambient temperature was controlled at 21–25°C, and the humidity was 60%–70%. Bedding was changed, and water and feed were added every 24 h.^23^ A TRPC3 agonist GSK1702934A was injected intraperitoneally into the neonatal rats in the control group and the BPD group at the concentration of 1 mg/kg^24^ on days 1, 3, 5, 7, 10, 12, and 14 after modeling to establish the control + TRPC3 agonist (control+) group and the BPD + TRPC3 agonist (BPD+) group.

### 2.2 Collection of tissue specimens

On days 1, 3, 5, 7, 10, and 14 after modeling, six rats in the control and BPD groups and six rats in the control + TRPC3 agonist and BPD + TRPC3 agonist groups were randomly selected and weighed; their weights and mortality were subsequently recorded. After intraperitoneal injection of 1.25% tribromoethanol, the chest was opened to expose the lungs, the left atrial appendage was cut open, and pulmonary circulation perfusion was performed with saline. Lung tissue was separated after the color of the lung changed from red to white. The left lung was placed in 4% paraformaldehyde for hematoxylin and eosin (HE) staining, and the right lung was frozen at −80°C for follow‐up experiments.

### 2.3 Hematoxylin and eosin staining

The left lung specimens soaked in 4% paraformaldehyde for more than 24 h were dehydrated. Next, paraffin sections were prepared and stained in HE dye solution. The stained specimens were observed and photographed under a microscope at 100× magnification.

### 2.4 Radial alveolar count

A vertical line was drawn from the center of the respiratory bronchioles to the nearest pleura or fibrous septum, and the number of alveolar counts on this line was defined as the radial alveolar count (RAC). The lung tissue structure of the HE‐stained sections was observed under a microscope at 100× magnification. Five field counts were taken from each section, with the average values taken as the RAC.

### 2.5 Quantitative real‐time PCR

We used Trizol reagent (Roche, Switzerland) to extract total RNA from the right lung tissue sample, and the RNA concentration was determined by spectrophotometry. The reverse‐transcription reaction was performed using the EvoScript Universal cDNA Master kit (Roche, Switzerland). The primers were designed by Sangon Biotech (Shanghai, China). The sequence of β‐actin primer was forward (5′–3′) GGTGTGATGGTGGGTATGGGT and reverse (5′–3′) CTGGGTCATCTTTTCACGGT; the sequence of TRPC3 primer was forward (5′–3′) GTGTCTGGTCGTGTTGGTCGTG and reverse (5′–3′) GATGATGAAGGAGGCAGCGTGAG; and the sequence of NF‐κBiz primer was forward (5′–3′) TCTGGAGGCAACGAACTATGATGG and reverse (5′–3′) AATGAGGCTGCTGGCTTCTCTG. The reaction condition was set up as follows: 95°C for 10 min; 95°C for 10 s, 65°C for 15 s, and 72°C for 20 s (40 cycles); and 65°C for 5 s, stopping at 95°C.

### 2.6 Western blot

The total protein of the right lung was extracted with a whole‐protein extraction kit (KeyGEN Biotech, Jiangsu, China), and the protein concentration was determined with the bicinchoninic acid protein quantification kit (KeyGEN Biotech, Jiangsu, China). We used a 7.5% PAGE gel rapid preparation kit (Yamei, Shanghai, China) to prepare concentrated glue and separation glue. The same amount of protein samples and markers was loaded into the gel hole. The amount of protein was 3–15 μg. Electrophoresis gel was maintained at a constant 150 V for 90 min, after which it was kept at 400 mA for 25 min to transfer the protein from the gel to the membrane. The blot was then covered in rapid sealing solution (Beyotime, Shanghai, China) and incubated with constant shaking for 10 min at room temperature. After washing the membrane with 1× Tris‐buffered saline with Tween (TBST) three times, for 10 min each time, we added primary antibodies against β‐actin (700068; Anti‐mouse; 1:500; Zen‐bioscience, Chengdu, China) and TRPC3 (77934; Anti‐rabbit; 1:1000; Cell Signaling Technology, Danvers, MA, USA) for incubation in a shaker at 4°C overnight. After washing the membrane in TBST three times, for 10 min each time, the rabbit secondary antibody (AS014; 1:2000; ABclonal, China) was incubated in a shaker at room temperature for 1 h. Then, after again washing the membrane in TBST three times, for 10 min each time, a chemiluminescent imaging system was used for assessment. TRPC3 was calculated as the ratio of the target protein band normalized with the β‐actin band gray value.

### 2.7 Tissue immunofluorescence

The paraffin‐embedded lung tissue sections were taken, and antigen retrieval was achieved with Tris‐EDTA buffer. A solution of 5% bovine serum albumin was used to incubate the samples for 1 h at room temperature. After washing the sections in TBST, we added TRPC3 primary antibody (NBP1‐70352; Anti‐goat; 1:500; Novus Biologicals, USA) for further incubation at 4°C overnight. Then, we added Cy3‐conjugated Donkey Anti‐Goat IgG (H + L) (SA00009‐3; 1:100; Proteintech, Wuhan, China) and 4,6‐diamidino‐2‐phenylindole (Beyotime, Shanghai, China) in the dark. Observations and photographs were taken with a Nikon C2+ confocal laser microscope at 200× magnification. ImageJ software was used for semiquantitative analysis of fluorescence intensity.

### 2.8 Statistical analysis

The experimental data were statistically analyzed using GraphPadPrism8.0.2 software and were evaluated using *t* test between two groups and multiple *t* test between groups. Differences with *p* values lower than 0.05 were considered significant.

## 3 RESULTS

### 3.1 Establishment of the hyperoxic lung injury model of bronchopulmonary dysplasia

**FIGURE 1:**
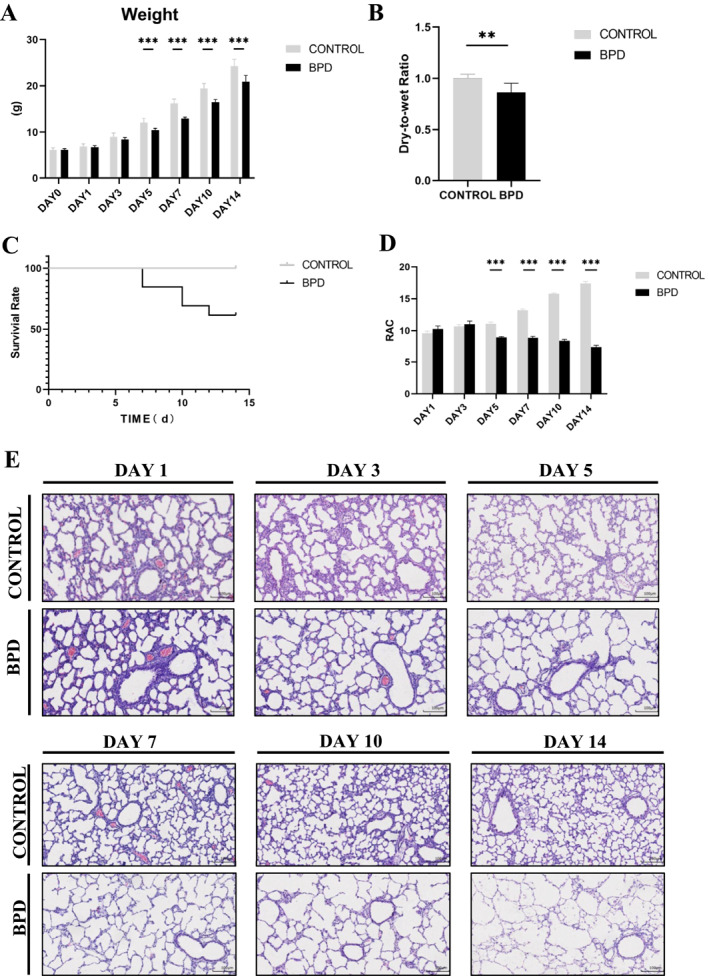
(A) Comparison of body weight between the control group (*n* = 8) and bronchopulmonary dysplasia (BPD) group (*n* = 8) at every time point. (B) The dry‐to‐wet lung ratio between the control group (*n* = 6) and the BPD group (*n* = 4) on day 14. (C) Survival curves of 0–14 days between control group (*n* = 13) and BPD group (*n* = 13). (D) Radial alveolar count (RAC) value of lung tissues in the BPD model. (E) Hematoxylin and eosin (HE) staining (100×) of lung tissue in the BPD model. (**p < 0.01, ***p < 0.001) Scale Bar = 100μm

At birth, body weight was similar in the control group and the BPD group (Figure 1A). While there were no significant intergroup differences on days 1 and 3, the body weight of the BPD group was significantly lower than that of the control group from day 5 onward (*p* < 0.01).

The number of deaths and general conditions of the neonatal rats in the control and BPD groups were recorded, and the survival curve was drawn (Figure 1C). No death occurred in the control group, and the rats responded well, grew normally, had rosy skin and smooth hair, and were able to open their eyes on day 14. In the BPD group, death began to occur from day 7 onward; a large number of deaths occurred on day 10; and the survival rate was approximately 60% on day 14. In addition, the BPD rats had poor response, decreased autonomic activity, weakened muscle tone, general tremor, retinopathy, decreased pain perception, increased respiratory rate, and signs of dyspnea such as cyanosis of mouth, nose, and limbs, and they were not able to open their eyes autonomously on day 14.

On day 14 after modeling, the lung dry‐to‐wet weight ratio was calculated (Figure 1B) and showed that the neonatal rats in the BPD group developed pulmonary edema.

Left lung tissues of the neonatal rats in the control and BPD groups were taken at every time point, and the lung morphological changes were observed by HE staining (Figure 1E). Specifically, with the increase in time, the lung tissue morphology of the control group became increasingly mature, the number of alveoli gradually increased, the volume gradually decreased, the thickness of pulmonary septum gradually decreased, the secondary septum gradually increased, and the alveolar ridge structure became more abundant and showed progressive development. Compared with the control group at the same time point, significant changes began to appear in the BPD group from day 5 onward. Namely, the number of alveoli decreased, the volume increased, the alveolar ridge and secondary septa were not obvious or even disappeared, and the number of pulmonary microvessels decreased. The changes in the BPD group on days 10 and 14 further expanded, the thickness of pulmonary septa was uneven, the alveolar structure was simplified, and the number of pulmonary microvessels decreased. A large number of inflammatory cells appeared.

We also determined the RAC values of the control group and the BPD group (Figure 1D). Namely, the RAC value of the control group gradually increased, indicating the gradual increase in the number of alveoli and gradual improvement in the lung tissue development in the control group. However, the RAC value of the BPD group was significantly lower than that of the control group (*p* < 0.01) and showed a downward trend, indicating the reduction in the number of alveoli and abnormal lung tissue development in the BPD group. The difference in the RAC values between the two groups might further explain the lung development disorder in the neonatal rat BPD model.

### 3.2 Expression of TRPC3 in the lungs of neonatal rats decreases under hyperoxia stimulation

**FIGURE 2:**
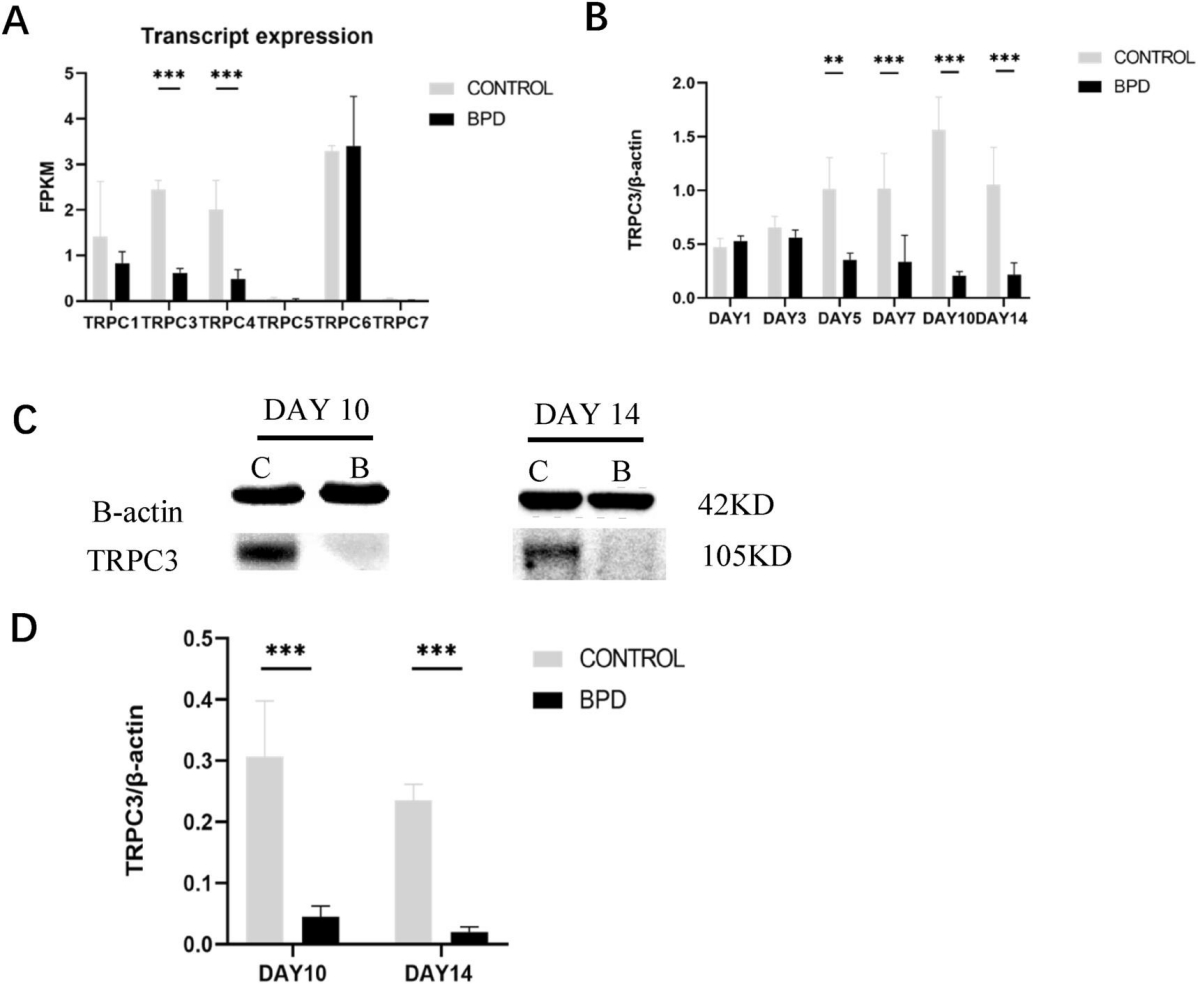
(A) Differential expression of transient receptor potential channel (TRPC) family members in the transcriptome sequencing. (B) The mRNA expression of transient receptor potential channel 3 (TRPC3) in lung tissues of the bronchopulmonary dysplasia (BPD) model. (C) Western blot (WB) band of TRPC3 in the lung tissues of the BPD model, with C representing the control group and B representing the BPD group. (D) Statistical analysis of TRPC3 protein expression in lung tissues. (control group: *n* = 4, BPD group: *n* = 4) (**p < 0.01, ***p < 0.001)

Three rats per group were selected on day 14 after modeling, and differentially expressed genes were obtained by reference transcriptomic sequencing. The absolute value of log2 (fc) ≥ 1 and *p* value < 0.05 were used as the screening criteria for the differentially expressed genes, and the results demonstrated that TRPC3 showed a downward trend in the BPD group compared with the control group (Figure 2A).

The pulmonary mRNA expression level of TRPC3 in the BPD model was detected by Quantitative real‐time PCR (qPCR) at every time point (Figure 2B). There was no significant intergroup difference in the mRNA expression level of TRPC3 on days 1 and 3, whereas the mRNA expression of TRPC3 in the BPD group was lower than that in the control group from day 5 onward (*p* < 0.01).

Western blot (WB) was applied to detect the protein expression level of TRPC3 in the lung tissues of the BPD model on days 10 and 14 (Figure 2C,D). The results showed that the protein level of TRPC3 in the BPD group was lower than that in the control group on days 10 and 14 (*p* < 0.01), which was consistent with the results of qPCR.

### 3.3 Verification of the localization and expression of TRPC3 in lung tissues of the bronchopulmonary dysplasia model by tissue immunofluorescence

**FIGURE 3:**
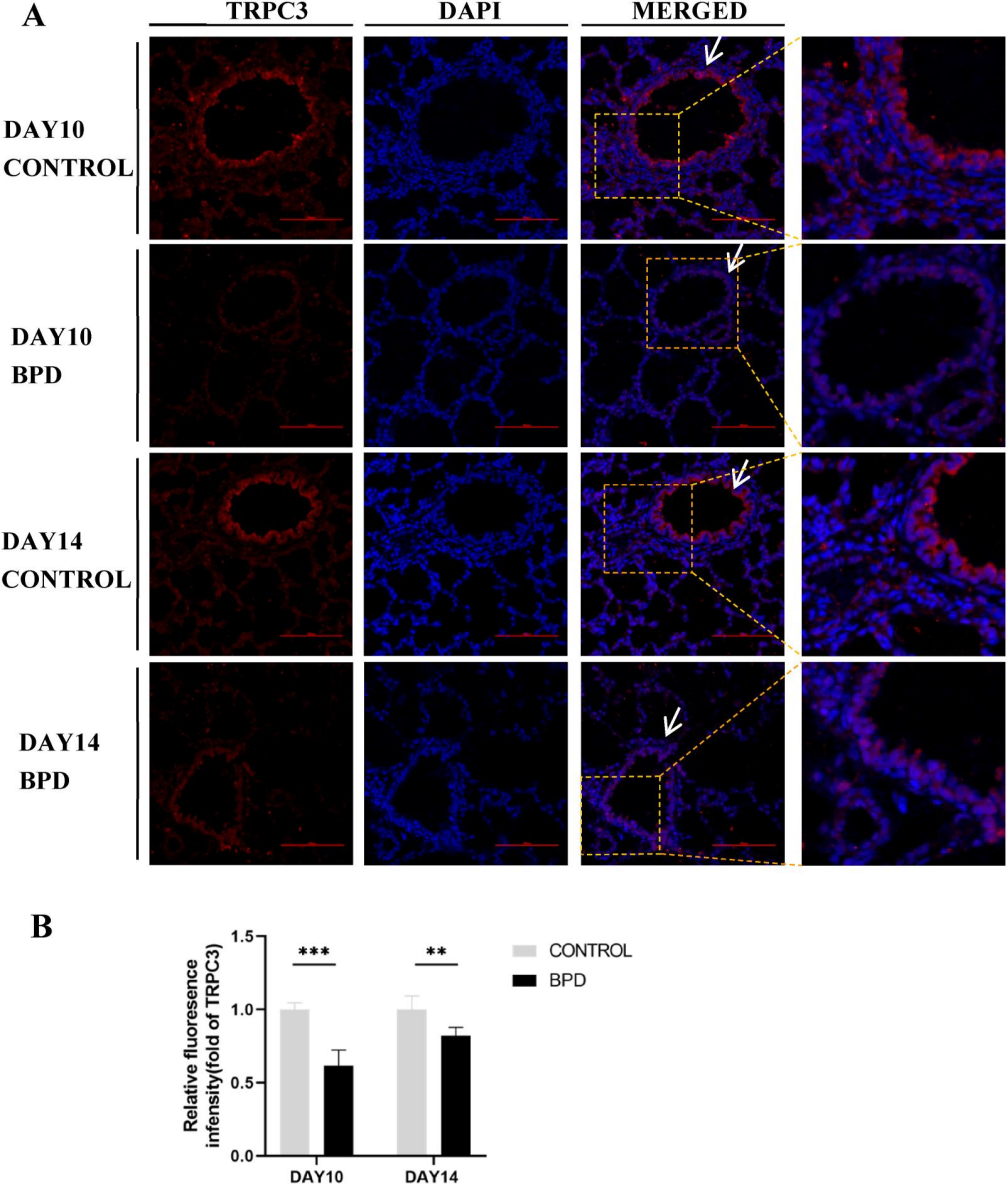
(A) Transient receptor potential channel 3 (TRPC3) immunofluorescence (200×) in lung tissue of bronchopulmonary dysplasia (BPD) model, TRPC3 (red), 4,6‐diamidino‐2‐phenylindole (blue). (B) Semi‐quantitative immunofluorescence analysis of TRPC3 in lung tissues of BPD model. (control group: *n* = 4, BPD group: *n* = 4) (**p < 0.01, ***p < 0.001) Scale Bar = 100μm

The paraffin‐embedded sections of left lung tissues of the neonatal rats in the control and BPD groups were taken for tissue immunofluorescence staining on days 10 and 14. TRPC3 was widely expressed on the cell membranes in the lungs, mainly in airways and pulmonary blood vessels (Figure 3A). The results of semiquantitative immunofluorescence analysis showed that the fluorescence intensity of TRPC3 in the BPD group was lower than that in the control group on days 10 and 14 (*p* < 0.01) (Figure 3B), which was consistent with the results of qPCR and WB.

### 3.4 Changes in phenotype, lung histomorphology, and lung development of the bronchopulmonary dysplasia model after TRPC3 stimulation

**FIGURE 4:**
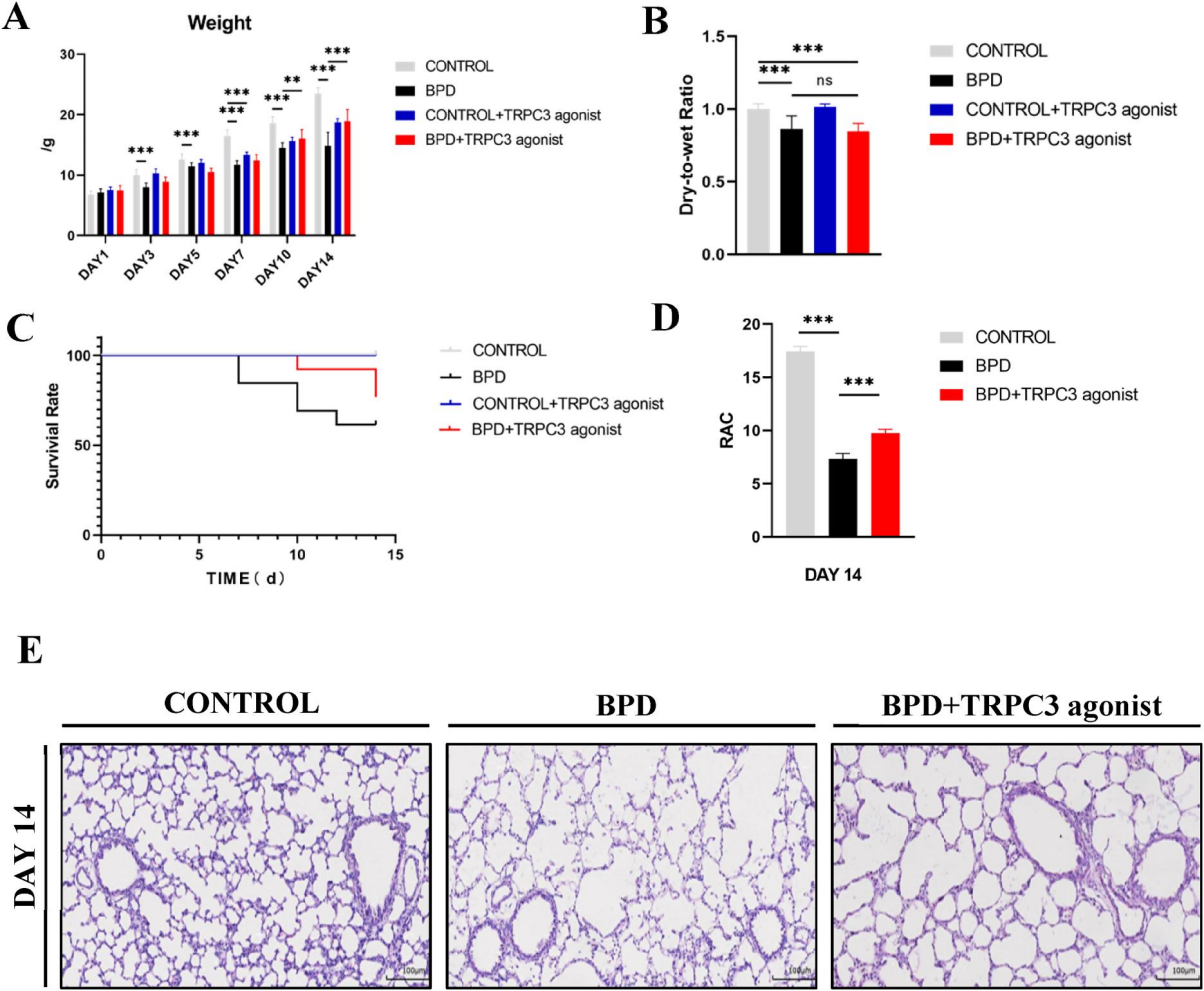
(A) Comparison of body weight at every time point in control group, control + group, bronchopulmonary dysplasia (BPD) group and BPD + group (every group: *n* = 8). (B) The lung dry‐to‐wet ratio of control group (*n* = 6), control + group (*n* = 6), BPD group (*n* = 4) and BPD + group (*n* = 6) on day 14. (C) Survival curves of 0–14 days control group, control + group, BPD group and BPD + group (every group: *n* = 13). (D) radial alveolar count (RAC) value of lung tissue in BPD + group. (E) Hematoxylin and eosin (HE) staining (100×) of lung tissues of BPD + group. (**p < 0.01, ***p < 0.001) Scale Bar = 100μm

The neonatal rats in the control group, the control + group, the BPD group, and the BPD + group were weighed and compared (Figure 4A). Compared with the control group, body weight of the control + group began to decrease from day 7 onward (*p* < 0.01). Compared with the BPD group, body weight of the BPD + group began to increase from day 10 onward (*p* < 0.01) and further increased on day 14 (*p* < 0.01), but it was still worse than that of the control group.

The death numbers and general conditions of the neonatal rats in the four groups were recorded, and their survival curves were drawn (Figure 4C). The results of the control group and the BPD group were the same as the results of the former experiment (Figure 1C). The results of the control + group were consistent with those of the control group. In the BPD + group, a small number of deaths occurred on day 10; death occurred before and after the restoration of normal oxygen on day 14; and the survival rate on day 14 was approximately 85%. Compared with the rats in the BPD group, those in the BPD + group had better response, increased autonomic activity, enhanced muscle tone, good response to pain, some improvement in cyanosis, and no retinopathy, but they were still not able to open their eyes autonomously on day 14.

The lung dry‐to‐wet weight ratio (Figure 4B) in the four groups was calculated. We found that there was no significant improvement in the BPD + group after injection of the TRPC3 agonist.

On day 14 after modeling, the paraffin‐embedded sections of the left lung tissue of the neonatal rats in the control group, the BPD group, and the BPD + group were subjected to HE staining (Figure 4E). Compared with the BPD group, the number of alveoli increased significantly, the volume decreased, the thickness of pulmonary septum increased, the number of pulmonary microvessels increased, and the infiltration of inflammatory cells decreased in the BPD + group, but the recovery degree of alveolar ridge structure and secondary septum was still lower than that of the control group.

The RAC values of the control group, the BPD group, and the BPD + group were measured and counted (Figure 4D). Compared with the BPD group, the number of alveoli increased and the abnormal development of the lung tissue improved in the BPD + group, but the recovery was not as good as that in the control group.

### 3.5 Changes of NF‐κB–related factors in the bronchopulmonary dysplasia model after activation of TRPC3

**FIGURE 5:**
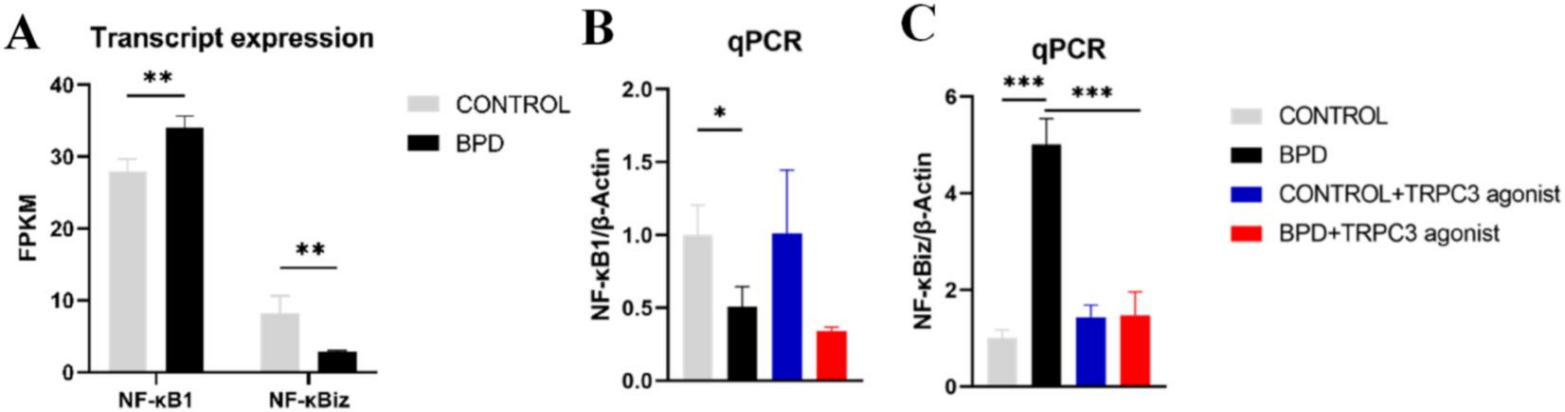
(A) Expression of NF‐κB1 and NF‐κBiz in the transcriptome sequencing. The mRNA expression of (B) NF‐κB1 and (C) NF‐κBiz in lung tissue of bronchopulmonary dysplasia (BPD) + group (every group: *n* = 4) (*p < 0.05, ***p < 0.001)

The transcriptome sequencing results (Figure 5A) suggested that TRPC might regulate downstream cytokines and transcription factors through the NF‐κB pathway. Therefore, qPCR was used to detect the changes in the expression of the NF‐κB pathway–related factors in the lung tissues of the BPD group on day 14 after TRPC3 agonist injection. Compared with the control group, the expression of NF‐κB1 decreased (*p* < 0.05) and the expression of NF‐κBiz significantly increased (*p* < 0.01) in the BPD group, while the expression of NF‐κBiz was inhibited after the activation of TRPC3. The expression of NF‐κBiz in the BPD + group decreased significantly (*p* < 0.01) (Figure 5B,C).

## 4 DISCUSSION

In recent years, the progress of perinatal medicine and the improvement of the neonatal monitoring level have improved the survival rate of premature infants, which has contributed to the rise in the incidence of BPD. Bronchopulmonary dysplasia is associated with neurodevelopmental abnormalities, retinopathy of prematurity, and long‐term respiratory complications in adulthood, seriously increasing the burden on individuals, families, and society. Thus far, there is no clear and effective targeted therapy to prevent and treat BPD. Some evidence supports the use of prenatal glucocorticoids, protective noninvasive ventilation, and early caffeine therapy, but none of the existing treatments could improve the long‐term prognosis of BPD significantly. It is important to explore the mechanism of lung injury repair in BPD to intervene in the course of BPD and to find effective treatments for BPD.

In this study, we adopted the classical modeling method^25^ to establish a BPD model with SD neonatal rats in a hyperoxic environment. We verified the successful establishment of the BPD model by comparing the body weight, survival curve, lung dry‐to‐wet weight ratio, HE staining, and RAC value between the control group and the BPD group. Transient receptor potential canonical channels are nonselective Ca^2+^ channels that are widely expressed in human and animal lungs. It is involved in oxidative stress response, airway mechanical pulling, and regulation of the integrity and permeability of the lung endothelial barrier. We thereby hypothesized that TRPC3 could play an important role in hyperoxia‐induced BPD. Our research revealed that the expression of TRPC3 gradually decreased with the increase in hyperoxia exposure time, which may be related to the reduction of the number of cells in lung tissue due to the simplification of alveolar and pulmonary blood vessels in BPD, and the decrease in Ca^2+^ influx caused by the decrease in cell proliferation and anti‐inflammatory response caused by the decrease of TRPC3 may aggravate the occurrence of BPD. Therefore, TRPC3 might be involved in the occurrence and development of BPD.

Ca^2+^ homeostasis plays an important role in maintaining the integrity of the lung endothelial barrier. Previous studies^26^ have shown that TRPC3 is expressed in pulmonary artery endothelial cells, and TRPC3 is a major nonselective cation channel in human ASMC, which is related to the proliferation of PASMC.^15^ Therefore, this study further analyzed the regulatory role of TRPC3 in BPD. After intraperitoneal injection of a TRPC3 agonist, the general condition, growth state, and lung development in the BPD + group significantly improved compared with the BPD group, but pulmonary edema and a small amount of inflammatory cell infiltration remained. This suggests that activation of TRPC3 may improve the progression of BPD.

Nuclear factor‐κB family transcription factors are widely expressed in humans and animals and are involved in regulating cell proliferation, survival, immunity, and inflammation.^27^ In normal cells, five subunits of NF‐κB (RelA/p65, RelB, c‐Rel, p50/NF‐κB1, and p52/NF‐κB2) form homo‐ or heterodimers that bind to typical inhibitory proteins (IκBα, IκBβ, and IκBε) and remain inactivated in the cytoplasmic state.^28^ Iκbζ, a transcription factor encoded by NF‐κBiz, is an atypical nuclear member of the IκB family and has a low expression level in most resting cells.^29^, ^30^ Oxidative stress and inflammation lead to the phosphorylation of typical IκB and the nuclear translocation of NF‐κB.^30^ In the nucleus, IκBζ can bind to p50 to enhance the transcription of NF‐κB and can also bind to p65 to inhibit the activity of NF‐κB,^30^ which is an important regulator of inflammation, cell proliferation, and survival.^31^

It has been demonstrated that inhibition of NF‐κB in the developing lung can disrupt angiogenesis and alveolar formation,^29^ and activation of NF‐κB has proinflammatory or anti‐inflammatory effects.^32^ Losef et al.^29^ found that blocking NF‐κB activity in 6‐day‐old neonatal mice could induce alveolar simplification similar to that in BPD and significantly reduce pulmonary capillary density, and NF‐κB can promote angiogenesis by regulating vascular endothelial growth factor. McKenna et al.^33^ found that sustained NF‐κB activation improved the survival of neonatal mice exposed to high oxygen levels and maintained lung development. Alvira et al.^32^ found that inhibition of NF‐κB activity in neonatal mice reduced apoptosis but increased inflammation, whereas activation of NF‐κB in neonatal mice lungs had anti‐inflammatory effects. The increase in intracellular Ca^2+^ concentration can lead to the activation of NF‐κB.^34^ Previous studies^35^ have demonstrated that the entry of Ca^2+^ through TRPC channels is a necessary condition for AMP‐activated protein kinase C to activate NF‐κB in endothelial cells. TRPC3 is involved in cytoplasmic Ca^2+^ elevation, activation of transcription factor NF‐κB, and cytokine upregulation.^36^ Oxidative stress and inflammation can lead to increased expression of NF‐κBiz and induce a variety of downstream molecules to participate in defense response.^37^ TRPC3‐mediated increase in Ca^2+^ signaling activates the transcription factor NF‐κB via the IκB, causing cell proliferation and leading to airway remodeling.^38^ Therefore, in this study, we hypothesized that TRPC3 might be involved in the pathogenesis of BPD through the NF‐κB signaling pathway. According to our results, under the condition of hyperoxia, the expression of TRPC3 in the BPD model decreases, the expression of NF‐κBiz increases, and the expression and activity of NF‐κB are reduced, which may inhibit cell proliferation and cause lung growth arrest. The expression of NF‐κBiz is inhibited after injection of the TRPC3 agonist. It may promote the activity of NF‐κB and improve the lung damage caused by hyperoxia. Therefore, the injection of calcium‐channel agonists such as TRPC3 agonist during the development of BPD may provide a potential therapeutic entry point for the prevention and treatment of BPD.

This study verified the expression of TRPC3 in the BPD model of hyperoxic lung injury for the first time. We also confirmed that TRPC3 was downregulated during the occurrence of BPD and that TRPC3 agonist was able to improve the disease progression of BPD, further confirming that TRPC3 participates in and regulates the occurrence of BPD through the NF‐κB signaling pathway. These findings provide a new direction and basis for understanding the pathogenesis of BPD and an important theoretical basis and reference for developing drug targets for the treatment of BPD.

However, there are some limitations to this study. For example, the lung morphological changes in the BPD + group after return to normoxia have not yet been detected, so it remains unknown whether the TRPC3 agonist can improve or delay the development of lung pathological changes in neonatal rats with BPD. We will further investigate the mechanism of TRPC3 and other TRPC channels in BPD by detecting TRPC3 expression levels and Ca^2+^ flow in vitro. The solution of these problems will help to further explain the occurrence of hyperoxic lung injury and the evolution of BPD, so as to provide potential therapeutic targets for clinical treatment of BPD.

## AUTHOR CONTRIBUTION

All authors contributed to the study conception and design. Material preparation, data collection and analysis were performed by Xingmeng Fu, Xiaoxia Gong and Tianyi Wu. The first draft of the manuscript was written by Xingmeng Fu and Chang Shu commented on previous versions of the manuscript. All authors read and approved the final manuscript.

## CONFLICT OF INTEREST STATEMENT

The authors have no relevant financial or non‐financial interests to disclose.

## ETHICS STATEMENT

Approval was granted by the Ethics Committee of Children's Hospital of Chongqing Medical University (No. CHCMU‐IACUC20220629002).

## CONSENT FOR PUBLICATION

Not applicable.

## Data Availability

The data that support the findings of this study are available from the corresponding author upon reasonable request.
